# Comparative Study of SERS-Spectra of NQ21 Peptide on Silver Particles and in Gold-Coated “Nanovoids”

**DOI:** 10.3390/bios13090895

**Published:** 2023-09-20

**Authors:** Siarhei Zavatski, Sergey Dubkov, Dmitry Gromov, Hanna Bandarenka

**Affiliations:** 1 Applied Plasmonic Laboratory, Belarusian State University of Informatics and Radioelectronics, 220013 Minsk, Belarus; s.zavatskij@bsuir.by; 2 Institute of Advanced Materials and Technologies, National Research University of Electronic Technology, Moscow 124498, Russia; svd@org.miet.ru (S.D.);; 3 Institute for Bionic Technologies and Engineering, I.M. Sechenov First Moscow State Medical University, Moscow 119435, Russia

**Keywords:** secondary structure, peptide, NQ21, surface-enhanced Raman scattering spectroscopy, silver particles, gold nanovoids

## Abstract

The NQ21 peptide has relatively recently attracted attention in the biomedical sphere due to its prospects for facilitating the engineering of the HIV1 vaccine and ELISA test. Today, there is still a need for a reliable and fast methodology that reveals the secondary structure of this analyte at the low concentrations conventionally used in vaccines and immunological assays. The present research determined the differences between the surface-enhanced Raman scattering (SERS) spectra of NQ21 peptide molecules adsorbed on solid SERS-active substrates depending on their geometry and composition. The ultimate goal of our research was to propose an algorithm and SERS-active material for structural analysis of peptides. Phosphate buffer solutions of the 30 µg/mL NQ21 peptide at different pH levels were used for the SERS measurements, with silver particles on mesoporous silicon and gold-coated “nanovoids” in macroporous silicon. The SERS analysis of the NQ21 peptide was carried out by collecting the SERS spectra maps. The map assessment with an originally developed algorithm resulted in defining the effect of the substrate on the secondary structure of the analyte molecules. Silver particles are recommended for peptide detection if it is not urgent to precisely reveal all the characteristic bands, because they provide greater enhancement but are accompanied by analyte destruction. If the goal is to carefully study the secondary structure and composition of the peptide, it is better to use SERS-active gold-coated “nanovoids”. Objective results can be obtained by collecting at least three 15 × 15 maps of the SERS spectra of a given peptide on substrates from different batches.

## 1. Introduction

A reliable express analysis of diverse proteomic analytes, solving the problem of the simultaneous detection, identification, and structural study of target substances at low concentrations, is still an unfulfilled task in many areas, including but not limited to medicine [1], biology [2], forensics [3], ecology [4], and pharmaceutics [5]. In recent years, interest has gradually been growing in revealing the secondary structure of proteins and peptides in trace amounts [6,7,8]. Many approaches can be used to study the secondary structure of proteomic analyte molecules. For instance, X-ray crystallography [9,10] and nuclear magnetic resonance spectroscopy [11,12] are traditionally exploited to achieve this goal. However, the short-peptide analysis offered by these methods is ineffective when a single sample contains various structural types of peptide or its oligomers are ordered in different ways. Circular dichroism (CD) spectroscopy is the most popular technique to overcome this hurdle [13], but the Protein Circular Dichroism Data Bank is not rich enough to confidently state that the most appropriate spectrum completely characterizes the previously unknown secondary structure of a new peptide [14,15]. In addition, the presence of tryptophan residues in a peptide can hinder the CD signal from its secondary structure [16,17]. Even ignoring all these shortcomings, we need to understand that the above-mentioned methods can hardly be used to study proteins and peptides at rather low concentrations, which are often required for vaccine or assay development [8,18,19].

In this case, a combination of optical techniques and nanotechnology can be used as a proof method, in particular, surface-enhanced Raman scattering (SERS) spectroscopy [20,21]. Spontaneous Raman spectroscopy has also been applied to investigate proteomic analytes [22,23], because Raman band positions in a spectrum are specific to each vibrational type of chemical bond in a molecule [24]. This peculiarity of Raman spectroscopy allows one to fingerprint molecules with polar covalent bonds. Raman spectroscopy results can be properly interpreted without reference spectra. At the same time, the method loses its sensitivity if the concentration of the analyte reaches a submolar level due to the narrow cross-section of the Raman scattering. A pronounced enhancement in the Raman signal is achieved when the molecules of the analyte are arranged near the surface of nanoparticles of noble metals (silver and gold are the most popular)—so-called SERS-active substrates [25,26]. Due to the surface plasmon resonance (SPR) arising in metallic nanoparticles upon electromagnetic irradiation by a laser, the number of Raman photons scattered from the molecules increases greatly, and the enhancement factor of the Raman signal can reach 10^6^ or more [27,28]. From the perspective of proteomic analyte study, SERS spectroscopy has many advantages: (i) often, there is no need for sample preparation or the preparation procedure is simple; (ii) the background from water and buffer solutions is not intensive enough to overlap with the analyte’s SERS spectrum; (iii) the luminescence from organic analytes is inhibited by metal particles; (iv) analysis can be quickly performed by laboratory staff that have no special skills in nanomaterials science, optics, or photonics. In general, the Raman signal enhancement provided by metallic nanostructures depends on such factors as the substrate nanotexture, the type of metal, the excitation wavelength and polarization, the shift of the Raman band in relation to the excitation light wavelength, the orientation of the bonds, the vibrations targeted for detection, the anharmonicity of oscillations during molecule adsorption on the substrate, and the nature of molecular binding to the substrate, to name a few. These particularities of SERS spectroscopy lead to some differences between the Raman and SERS spectra of the same analyte [29], but they can be applied in a positive way, e.g., for the determination of the orientation [30] or adsorption sites [7] of molecules on metallic surfaces. During the last decade, the SERS spectroscopy of proteins/peptides has progressed substantially and has been applied not only for the study of the position and intensity of Raman bands of numerous analytes [22,23] but also for the detection of biomarkers via peptide recognition [31], peptide-antibody assays [32], distinguishing between different peptide mutation states [33], and the selection of peptide affinity for the detection of toxins [34]. Considering the study of the secondary structure of proteins/peptides, the amide I and amide III bands in the Raman spectrum play the role of indicators [22,35,36]. In principle, finding the position of amide I is enough to identify the alpha-helix, beta-sheet, mixed, or random coil structure of the analyte [37]. However, if the analyte is adsorbed on the surface of metallic nanoparticles, the chemical bonds of some molecules associated with amide I (−C=O) are located rather far from spots of the SPR-induced enhancement of the electromagnetic field due to bulky side chains [38]. Consequently, the amide I band cannot appear in each SERS spectrum. Therefore, to obtain reliable results on the secondary structure, there is a need to find several appropriate SERS patterns of an analyte molecule containing the amide I band when a SERS spectra array is collected. The number of SERS measurements at different points can exceed several hundred [39]; thus, the entire recording process, including the recognition and selection of informative spectra, usually results in a lengthy procedure.

In [8], a structural study of the NQ21 peptide including information on the molecular weight of the peptide depending on the pH and base number, which corresponded to a less mutable part of the first conserved region of envelope protein gp120 of human immunodeficiency virus type 1 (HIV1), was reported. The interest in the NQ21 peptide is caused by its prospects for the development of a synthetic vaccine against HIV1 without conjugation to a carrier protein [39,40]. The results of the SERS measurements, which showed the domination of the alpha-helix structure, were verified by CD spectroscopy [8]. However, a detailed description of a methodology for SERS analysis including optimal measurement regimes and SERS-active materials was not provided in [8] or other similar works [22,38]. The development of such a methodology could be very useful to speed up and facilitate the routine study of the secondary structure of proteins/peptides.

In this study, we performed multiple systematic SERS measurements of the NQ21 peptide at different pH levels using two types of SERS-active substrates. The first one was a film composed of silver nanoparticles on mesoporous silicon reported to be sensitive to a femtomolar concentration of analytes, including proteins [41]. The surface of the silver-coated mesoporous silicon substrate was also shown to be negatively charged, which prevented the denaturation of the proteomic analyte [41]. It was selected because we aimed to study a small amount of the peptide, to which spontaneous Raman spectroscopy would be insensitive [8]. The other substrate was a conformal gold film coating the surface of macropores in silicon [42], forming so-called “antinanoparticles” or “nanovoids” [43,44,45]. Such a morphology is attractive for investigating proteomic analytes because “antinanoparticles” promote the SERS effect via the multiple interreflection of excitation light inside the void [43,46]. As a result, the reflected electromagnetic field forms a 3D cloud, which is expected to uniformly excite all the bonds in the analyte molecule in contrast to small hotspots between plasmonic nanoparticles [47]. In addition, gold nanostructures are SERS-active when subjected to an infrared laser whose wavelength lies in an optical transition window range [48]. The precise management of the morphology of the SERS-active substrates in our research was achieved by sculpting a silicon template used for the deposition of silver and gold films. Spectra processing was carried out with a newly developed algorithm for finding the spectra containing the amide I band. During this research, the optimal size of the SERS map (the number of points on the spectra periodically recorded from the specific area on the SERS-active substrate) sufficient for the definition of the secondary structure of the peptide was determined. We showed that the SERS measurements with the gold substrate provided a two- to three-fold increase in the number of peptide SERS spectra with an amide I band and nearly completely prevented analyte burning in comparison with the silver particles. However, the silver substrates provided a more prominent SERS signal intensity from the analyte molecules. An explanation of the observed effects was proposed considering the molecules’ locations and the electromagnetic field distribution on the metal coatings.

## 2. Materials and Methods

### 2.1. Synthesis and Preparation of Peptide Solutions

The NQ21 vaccine peptide designed by Khrustalev V.V. was kindly provided in the form of phosphate buffer solutions (PBSs) with specific pH levels. Briefly, a “Symphony” automatic solid-phase peptide synthesizer (Protein Technologies, Inc., Manchester, United Kindom) was used to synthesize the NQ21 peptide. The quality of the peptide was controlled with an Agilent 1200 high-performance liquid chromatographer (Agilent Technologies, Inc., Santa Clara, CA, USA) and a Shimadzu LCMS-2010 mass spectrometer (Shimadzu Corp., Kyoto, Japan).

SERS analysis was performed to study the NQ21 peptide (at a concentration of 30 µg/mL) in water solutions with 0.01 M phosphate buffer. The NQ21 solutions had variable pH levels of 6.8, 7.4, and 8.0.

### 2.2. Fabrication and Characterization of SERS-Active Substrates

SERS-active substrates were fabricated using a two-step process. Firstly, monocrystalline silicon wafers were electrochemically etched to form porous silicon. The etching was carried out according to the procedures described in [49] for mesoporous layers and [50] for macroporous layers with slight modifications. The potentiostat/galvanostat AUTOLAB PGSTAT302n (Metrohm, Herisau, Switzerland) was used to conduct the electrochemical processes. Then, the metal coatings were formed by immersion deposition on meso- and macroporous silicon. The silver particles were grown in a 3 mM AgNO_3_ aqueous solution mixed with C_2_H_5_OH at a ratio of 99:1 (vol. %), as reported in [51]. The mesoporous silicon samples were immersed in this solution for 20 min upon continuous ultraviolet irradiation at a power of 8 W. The gold film was fabricated by immersing the macroporous silicon in 0.01 M Kau (CN)_2_ and 0.15 M HF (45%) for 40 min at 45 °C [42]. The silver- and gold-coated substrates were then thoroughly rinsed with the deionized water and ethanol and air-dried.

The structure and composition of the metallized porous silicon samples were studied with a Hitachi 4800 scanning electron microscope (SEM) (Hitachi, Tokyo, Japan) and DRON-3 X-ray diffractometer (XRD) (Burevestnik, Moscow, Russia) under CuK_α_ radiation and an X-ray wavelength λ = 0.15406 nm.

### 2.3. SERS Measurements

Just-prepared SERS-active substrates were incubated in R6G solution (to test the SERS-activity) and buffer solutions with and without peptide (to reveal if there was any contribution from the buffer solution to the SERS spectra) for 2 h. The spontaneous Raman spectroscopy of the peptides on fresh porous silicon (peptides were adsorbed on the porous silicon samples over 2 h) and a glass plate (peptides were drop-deposited) was also carried out to exclude any possibility of obtaining spectra without surface enhancement promoted by metallic nanostructures. A Confotec NR500 3D scanning confocal Raman microscope (SOL instruments, Minsk, Belarus) was used to perform SERS analysis. The measurements were achieved using a 100× objective with a 633 nm laser (~800 nm spot diameter) for the silvered substrates and a 40× objective with a 785 nm laser (~1200 nm spot diameter) for the gold-coated substrates. The SPR band positions for the silver and gold substrates were studied in [52] and [42], respectively. The polydisperse silver particles possessed the SPR band in the blue range, but it broadened to the red wavelengths. The gold-coated macroporous silicon was characterized by the first prominent SPR band in the green range, but the second one belonged to the near-IR range. Therefore, the wavelengths of both lasers selected for the SERS analysis overlapped with the SPR bands. The power of the 633 nm and 785 nm laser beams passed through the objective was 1.99 mW and 13.67 mW, respectively. The 633 nm laser power decreased from an initial 4.66 mW value to avoid analyte burning. The uniformity of the SERS signal intensity from the 10^−6^ M R6G analyte was evaluated through the collection of 100 spectra on the 100 µm^2^ spot of the substrates. Peptides from each solution were studied three times by the collection of 15 × 15 SERS spectra maps for the metal-coated substrates from different batches. The signal collection time was 2 s for the silvered substrates and 1 s for the gold-coated ones. Each spectrum was normalized and aligned in relation to the silicon band position at 520 cm^−1^ that appeared because of the underlaying monocrystalline silicon.

### 2.4. Algorithm of SERS Spectra Analysis

During this study, we developed an algorithm to provide a reliable analysis of the SERS spectra arrays that revealed all the patterns containing the amide I band. This algorithm excluded the spectra of amorphous carbon, which appeared if the analyte molecules were burned due to the effect of the combined electromagnetic fields from the laser and metallic nanostructures. As was mentioned in the Section 1, the conformation of the analyte molecule could be changed on the SERS substrate. To confidently avoid the selection of the peptide spectra with a changed structure, we took into account additional bands (Trp, C_α_-H, and N-C_α_-C) that are typical of the Raman spectra of proteins/peptides [9]. A detailed description and flowchart of this algorithm is presented in Figure S1.

## 3. Results and Discussion

### 3.1. Characterization of SERS-Active Substrates

Since the discovery of the SERS effect, a hundred different types of SERS-active substrate have been developed. In general, they are divided into liquid (metallic nanoparticles in solution) and solid (ordered arrays of metallic nanoparticles on a planar substrate) substrates [53]. Although liquid substrates provide a higher enhancement factor, here we used solid SERS-active materials because of their better reproducibility and usability. Substrates fabricated by the immersion deposition of noble metals on porous silicon were selected. This porous template is very attractive as a base for SERS-active substrates due to its simple formation process, easily controllable structure, rich morphological family, and the ability of ions of noble metals to be reduced on the silicon surface via fast and cost-effective immersion deposition. Thus, it is possible to easily manage the geometry and sizes of the deposited metallic nanostructures to adapt them for the SERS measurement of a given analyte in the absence of complicated nanotechnology equipment.

**Figure 1:**
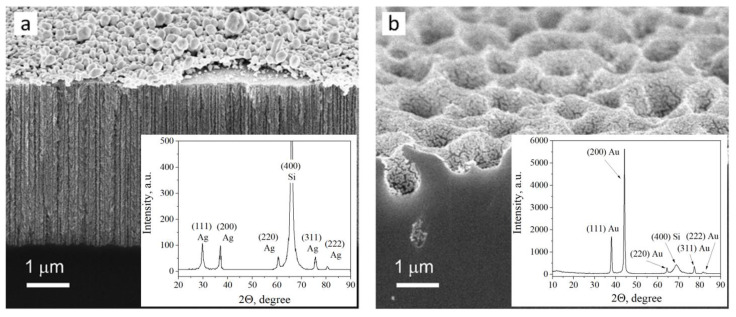
SEM cross-section images and XRD patterns of the SERS-active substrates based on (**a**) the silver-coated mesoporous silicon and (**b**) the gold-coated macroporous silicon.

To develop methodology for the SERS measurement of the NQ21 peptide, we started using substrates based on mesoporous silicon coated with silver particles. These substrates have already been reported as stable SERS-active materials that provide weak spot-to-spot and sample-to-sample deviations in the SERS signal intensity [54], a high enhancement factor [55], a femtomolar detection limit, and the ability to be used with the red excitation wavelength [52]. The last feature is very attractive for the study of proteins/peptides because silver nanostructures usually demonstrate SERS-activity under blue laser excitation. Joint effects from d-metal and a blue excitation wavelength can lead to the denaturation of these molecules. Figure 1a shows an SEM image and XRD pattern of the silver-coated mesoporous silicon used in the present work. The XRD analysis revealed that the silver coating had a polycrystalline nature. The silver was deposited as a quasi-continuous film containing particles whose sizes varied from a few to several hundred nanometers. In more detail, a bimodal size distribution of the silver particles was observed: the first size range was for particles below 120 nm, while the second was for larger particles [52]. The mesoporous silicon was characterized by a mean pore diameter of 50 ± 20 nm and a 5 ± 0.5 µm thickness. The effect of “bottle-neck” pore entrances prevented the significant penetration of silver particles into the porous layer, so the metallic coating was located on the external surface of the porous template. Specifically, the broad size distribution of the silver particles caused diverse plasmonic oscillation modes, which led to an extension of the SPR band to the long wavelength region of the visible spectrum and resulting SERS-activity at the red laser. During the peptide adsorption on the silver-coated substrate, the molecules were located between the silver particles. The Raman signal from the molecules was expected to be enhanced due to the overlap of the electromagnetic fields from different particles. The highest SERS intensity is typical for 3–10 nm gaps (“hotspots”) between metallic nanostructures [46]. A shorter gap can cause undesirable side effects due to electron transitions, while a wider gap is not sufficient for field interaction from the neighboring particles. In our case, the required distance and size of the silver particles were defined by the specific morphology of the external surface of the mesoporous silicon.

The idea of using a new SERS-active substrate based on gold-coated macroporous silicon was born during the first stage of the SERS measurements of the NQ21 peptide and is described in detail in the following subsection. In general, there was a need to provide conditions for the quasi-uniform stimulation of vibrations for all the bonds of the analyte molecules in an electromagnetic field and to completely avoid peptide denaturation. To achieve this objective, we used a macroporous silicon template, whose pores had a diameter and depth that varied in the range of 0.5 to 1.5 µm. Such dimensions are typical for plasmonic “nanovoids”, in which an electromagnetic field can be located inside the cavity of a metallic film due to multiple bounces of light [43]. In the present work, the macropores in the silicon were covered with a thin plasmonic layer composed of polycrystalline gold, as revealed by SEM and XRD (Figure 1b). We selected gold as the SERS-active metal because of its inertness and ability to enhance the Raman signal upon near-infrared irradiation. The gold nanoparticle diameters varied from 20 to 80 nm [42].

**Figure 2:**
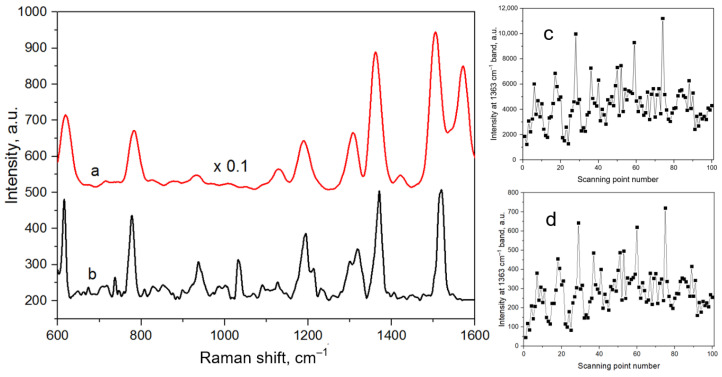
The mean SERS spectra of the R6G molecules (10^−6^ M) and SERS signal intensity of the 1363 cm^−1^ band at different scanning points: (**a**,**c**) on silver-coated mesoporous silicon at the 633 nm excitation wavelength and (**b**,**d**) on gold-coated macroporous silicon at the 785 nm excitation wavelength.

Figure 2 shows the SERS spectra of 10^−6^ M R6G adsorbed on the silver- and gold-coated porous silicon. Both samples demonstrated SERS-activity. Raman bands were observed at 613 cm^−1^ (C-C-C ring ip bend); 772 cm^−1^ (out-of-plane bend); 1185 cm^−1^ (C-H ip bend); 1311 cm^−1^ (C-O-C stretching); and 1363, 1510, 1575, and 1650 cm^−1^ (aromatic C–C stretching). The positions of these bands were in a good agreement with those of the SERS and Raman spectra of R6G reported elsewhere [56,57]. The SERS spectrum of R6G on the silver particles was registered using a 633 nm laser and showed activity one order of magnitude higher than that collected on the gold substrate with a 785 nm laser. The standard deviation of the SERS intensity at the 1363 cm^−1^ band was calculated using the data shown in Figure 2c,d in Origin 2018 to define the signal uniformity. The silver-coated mesoporous silicon provided a standard deviation of approx. 1677 a.u., while that of the gold-coated macroporous silicon was approx. 112 a.u. At the same time, we observed a prominent band at around 1584 cm^−1^ in the SERS spectrum related to the silvered substrate, which corresponded to amorphous carbon and appeared due to analyte burning. It should be noted that in cases of some organic analytes, it is undesirable to use substrates that cause an enormous increase in the electromagnetic field, which are usually selected to achieve a maximum enhancement factor, because this can lead to a complete change in the spectrum of an initial molecule.

Therefore, we fabricated two types of solid SERS-active substrates: (i) silver particles, which in the case of large molecules were expected to provide a substantial increase in the Raman signal from the chemical bonds that were located in “hotspots” but could simultaneously cause partial analyte destruction; and (ii) gold “nanovoids”, with which the enhancement in the Raman signal would be lower, but all the bonds of large molecules could be uniformly excited by an internal electromagnetic field in the metallized cavity, and the burning of the analyte would not take a place.

### 3.2. SERS Spectroscopy of the NQ21 Peptide

Studies of different peptides by SERS spectroscopy with silver- and gold-based substrates have been reported before [7,30,38]. The partial suppression of some Raman bands (e.g., the amide I band) in peptides with bulky side bonds has been observed. This phenomenon has been explained by the substantial distance between the internal molecule bonds and the surface of the metallic nanostructures. In [7], it was revealed that peptides containing tryptophan were anchored to the surface of gold nanoparticles via an indole ring, while the adsorption sites of tryptophan and its derivatives on silver nanoparticles were more variable and could be carboxylate and amino features. To find regularities in the SERS spectra of the NQ21 peptide and to develop a methodology for the SERS analysis of peptides, we performed a two-stage study.

**Figure 3:**
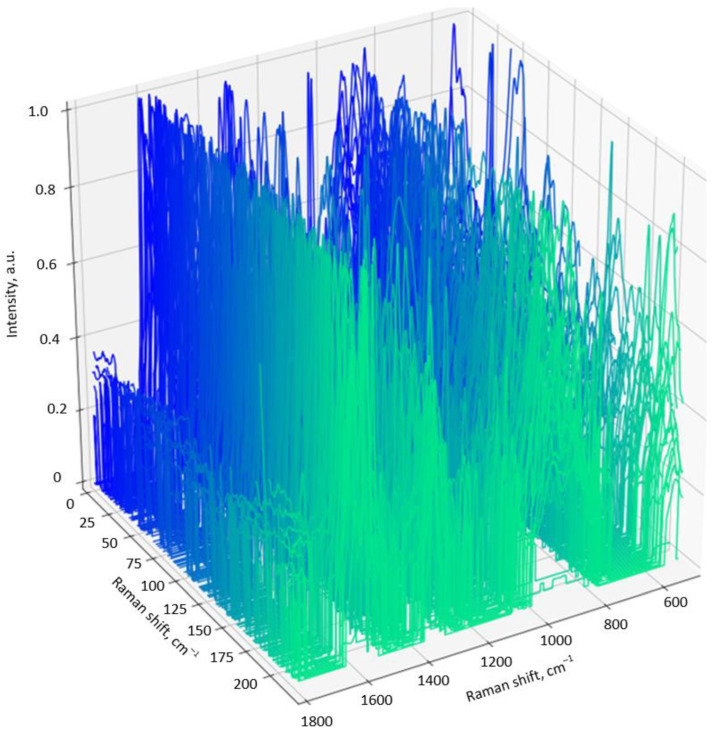
SERS-spectra of the NQ21 peptide adsorbed from solution with pH = 6.8 on the silver-coated mesoporous silicon.

**Figure 4:**
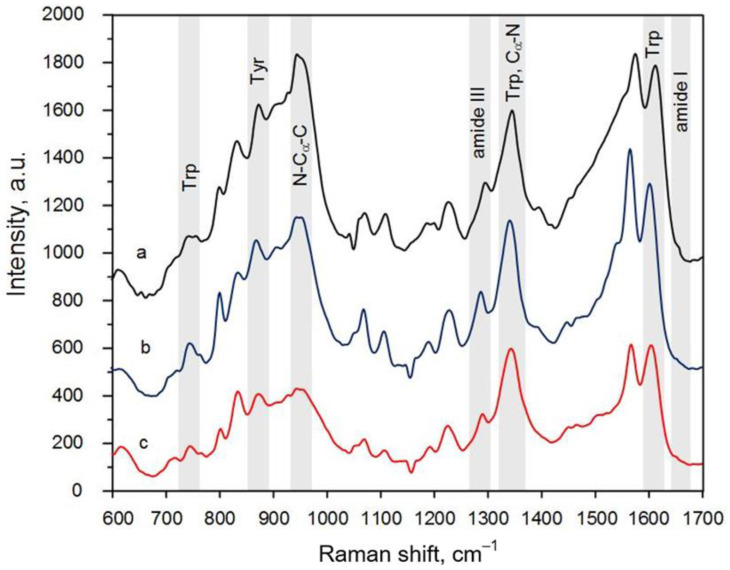
The mean SERS spectra of the NQ21 peptide adsorbed on the silver-coated mesoporous silicon from the solutions with a pH equal to (**a**) 6.8, (**b**) 7.4, and (**c**) 8. Each mean SERS spectrum was built from three sets of 225 SERS spectra collected from the silvered substrates of different batches.

Firstly, three maps of the peptide SERS spectra for different pH levels were collected. Each map contained 225 spectra that were collected with a 1 µm step. An example of the SERS spectra array for the peptide adsorbed on the silver-based substrate from a solution with pH = 6.8 is depicted in Figure 3. This view of the SERS spectra array was typical for all the SERS maps. It can be clearly seen that the patterns of each spectrum were not completely repeatable in terms of the intensity, position, and number of Raman bands. In general, this was caused by the different orientations of the chemical bonds, the anharmonicity of oscillations during adsorption on the substrate, chemical binding to the silver particles, and the denaturation of peptides. However, the mean spectra for all the SERS maps were characterized by the same set of bands, showing the reliability of the measurements (Figure 4). The following bands could be seen in the SERS spectra: 1610 cm^−1^ (Trp residue), 1405 cm^−1^ (–CH_3_, def. from side chains of hydrophobic residues), 1285 cm^−1^ (amide III), 945 cm^−1^ (N–C_α_–C), 859 cm^−1^ (asymmetric C-C-S stretching of Met side chains), and 754–758 cm^−1^ (Trp residues) [22].

Unfortunately, the main band of interest (amide I) can hardly be recognized in the spectra in Figure 4. Each map contained just a few SERS spectra with this band, which did not significantly contribute to the averaged result. Figure S2 in the Supplementary Materials together with Table 1 shows the average SERS spectra of the peptide for each map.

We divided the spectra into those that contained an amide I band and those without one. Considering SET 1 in Figure S2, we see that all the spectra with an amide I band at pH = 6.8 were two to three times less intensive than those at the higher pH. This could have been caused by the pH-dependent charge of the peptide molecules defining their position in relation to the substrate. Some SERS spectra had a band at 1449–1461 cm^−1^ (C-H, def.). In these cases, C-H bonds were located in the “hotspots”. Almost all the maps contained SERS spectra of amorphous carbon, signifying analyte burning. One can see intensive bands at 1360–1380 cm^−1^ and 1580–1590 cm^−1^ that were also associated with amorphous carbon. These relatively broad bands nearly completely hid the regions of the Trp, Ca-H, and indole ring bands, complicating objective spectra processing.

**Figure 5:**
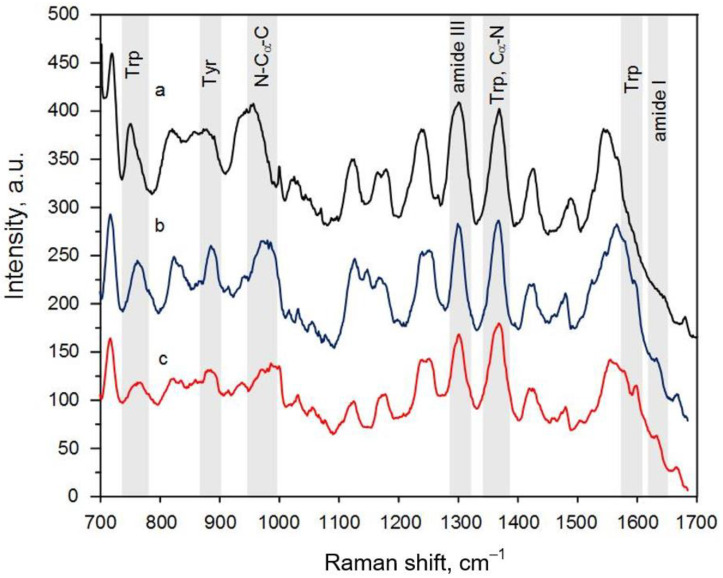
The mean SERS spectra of the NQ21 peptide adsorbed on gold-coated macroporous silicon from the solutions with a pH equal to (**a**) 6.8, (**b**) 7.4, and (**c**) 8. Each mean SERS spectrum was built from three sets of 225 SERS spectra collected from gold-coated substrates of different batches.

The SERS measurements made with the gold “nanovoids” resulted in the same set of Raman bands as in the case of the silver particles but with a modest enhancement (Figure 5). The SERS intensity of the peptide spectra on the gold substrate was about three times lower compared to those collected on the silver particles. However, all the bands could still be easily recognized in the peptide spectra. All averaged maps for the gold samples are also presented in Figure S3. It can be clearly seen that the deviation in the intensity of different Raman bands in the case of gold was not as broad as for the silver substrates, i.e., the vibrations were stimulated more uniformly for the side and internal chemical bonds compared to during the measurements with the silver particles. This likely took place due to the localization of the majority of the peptide molecules in the vast electromagnetic field inside the gold “nanovoids”. The spectrum with a low intensity (Figure S3, SET 1, pH = 7.4, second spectrum) was probably obtained in the area between the “nanovoids”, where the signal enhancement was insignificant. The amide I band was observed more often, because the electromagnetic field induced all the vibrational bonds of more peptide molecules than in the case of the silver particles. No amorphous carbon spectra were registered on the gold substrates. Thus, analyte burning was not proved. It should also be noted that the SERS spectra obtained on the gold substrate had a prominent band for the indole ring (1552–1557 cm^−1^). This proved the finding of [22] regarding the domination of adsorption sites of Trp-containing peptides on gold nanostructures via the indole feature.

**Figure 6:**
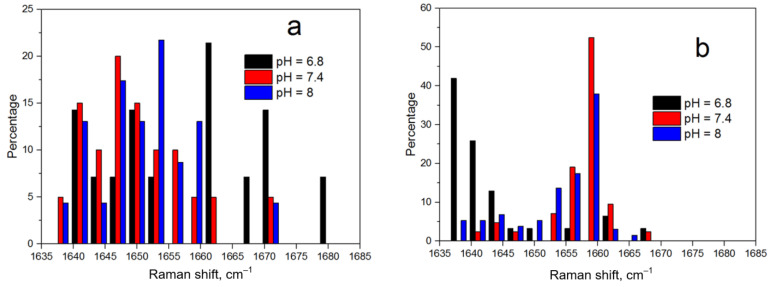
Histograms of the distribution of the amide I band position in the SERS spectra of the NQ21 peptide on (**a**) silver-coated mesoporous silicon and (**b**) gold-coated macroporous silicon.

To study in more detail the secondary structure of the NQ21 peptide, histograms of the distribution of the amide I band positions were built (Figure 6).

The SERS analysis with both silver and gold substrates showed that an alpha-helix structure dominated in the NQ21 peptide. This conclusion arose from the more frequent localization of histogram bands in the region of wavenumbers characteristic of an alpha helix, i.e., from 1637 to 1663 cm^−1^. However, no prominent regularities in the behavior of the amide I band according to the pH level were observed for the peptide SERS spectra registered on the silver particles. In addition, the corresponding histogram (Figure 6a) showed a tendency towards a mixed or random coil structure, especially for pH = 6.8, when black histogram lines could be seen near 1680 cm^−1^. This may have been caused by the partial denaturation of the NQ21 peptide on the silver particles, which was slightly inhibited in solutions with a pH > 7.4 due to the passivation of the SERS-active substrate through silver surface oxidation. Changes in the surface composition of the silver particles induced by sulfidation and/or oxidation could seriously affect the SERS-activity, e.g., suppressing it. The SERS spectra of NQ21 molecules collected from the silver-coated mesoporous silicon were characterized by a gradual decrease in the signal intensity when the pH level rose from 6.8 to 8.0, which was generally accepted to be caused by appearance of oxidation species. For example, the SERS intensity at the 945 cm^−1^ band (N–C_α_–C) decreased from ~1000 a.u. at pH = 6.8 via ~700 a.u. at pH = 7.4 to ~370 a.u. at pH = 8.0. The same behavior was typical for other characteristic bands. The SERS measurements with the gold “nanovoids” showed that the position of the amide I band shifted from 1638 cm^−1^ to a higher wavenumber (approx. 1663 cm^−1^) with a pH level increase but was still typical of an alpha-helix structure. The percentage of the SERS spectra containing the amide I band at a position related to beta-sheets (between 1665 and 1670 cm^−1^) was negligible and did not exceed 2–3%. The position of the amide III band in the averaged spectra within the range of 1270–1300 cm^−1^ also favored the prevalence of an alpha-helix structure in the NQ21 peptide [22].

**Figure 7:**
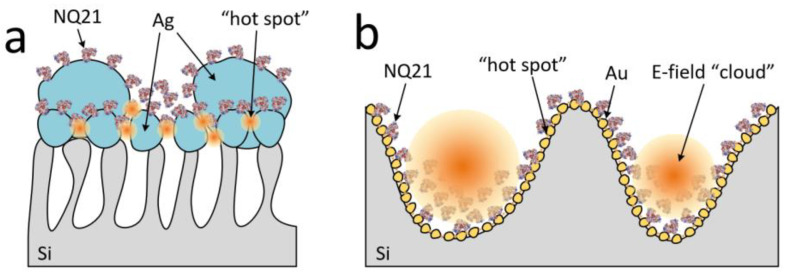
Scheme showing the anchoring configurations of NQ21 on the surfaces of (**a**) silver particles on mesoporous silicon and (**b**) gold “nanovoids” in macroporous silicon.

The pore size and distribution in the mesoporous silicon did not affect the arrangement of the peptide molecules (Figure 7a). This was caused by the complete coverage of the mesoporous surface with silver particles. The peptide molecules were adsorbed on the surface of the silver particles as a layer and were accumulated in the spaces between the large particles. The surface of the gold nanoparticles was also coated with the peptide layer, but the “nanovoids” or pores were filled with peptide molecules (Figure 7b). The silver particles did not provide a prominent SERS signal from the amide I part, i.e., its bonds were far from the “hotspots” on the substrate. Otherwise, characteristic bands of tryptophan were well-defined, including the indole ring, which meant that the molecules were located on the SERS-active surface near this part. In contrast, gold “nanovoids” provided an SERS signal from all the main bands of the peptide. This proved the existence of an overlap between the peptide molecules and the 3D electromagnetic field (E-field) induced by the incident light interreflection inside the void coated with gold nanoparticles. At the same time, we observed a more intensive SERS signal from the indole ring bonds, which indicated the adsorption of the peptide via this part of the molecule, enhancing its signature in the “hotspots” between the gold nanoparticles, as schematically represented in Figure 7b.

In general, the SERS measurements of the NQ21 peptide carried out with the silver particles and gold “nanovoids” allowed the collection of SERS spectra maps, whose assessment with the proposed algorithm resulted in repeatable mean SERS spectra and revealed the secondary structure of the analyte. However, using the gold-coated substrates led to more reliable SERS analysis data on the peptide’s secondary structure with less deviation in the band intensity, the absence of a band signifying analyte destruction, and the presence of an amide I band in more cases.

## 4. Conclusions

During this work, we carefully investigated the NQ21 peptide by SERS spectroscopy with solid SERS-active substrates based on silver particles on mesoporous silicon and gold “nanovoids” in macroporous silicon. This allowed us to achieve our research goal of developing a reliable methodology for studying peptides’ secondary structure by SERS spectroscopy. Silver particles are recommended for the detection of peptides if it is not urgent to precisely reveal all the characteristic bands. The silver-coated mesoporous silicon provided better enhancement, but the SERS measurements were accompanied by partial analyte destruction. If the goal is to carefully study the secondary structure and composition of a peptide, it is better to use SERS-active gold “nanovoids”. Objective results can be obtained by collecting at least three 15 × 15 maps of the SERS spectra of a given peptide on substrates from different batches. Further research will be devoted to testing the proposed methodology with other peptides and proteins, an experimental and theoretical study of the electromagnetic field distribution between silver particles and inside gold “nanovoids”, and the optimization of their structure to meet the strict requirements for the analysis of most proteins and peptides.

## Figures and Tables

**Table 1:** Statistics of the SERS spectra for the NQ21 peptide.

Type of SERS-Active Substrate	Type of SERS Spectrum	pH Level								
		6.8			7.4			8		
Silver particles	Good *	212	219	219	217	213	220	215	220	214
	Not defined **	3	0	0	0	2	0	1	0	0
	Good with amide I	6	4	4	7	9	4	8	5	11
	Amorphous carbon	4	2	2	1	1	1	1	0	0
Gold “nanovoids”	Good	209	198	205	173	190	192	112	200	197
	Not defined	1	11	6	40	2	5	1	4	3
	Good with amide I	15	16	14	12	32	28	112	21	25
	Amorphous carbon	0	0	0	0	0	0	0	0	0

* Spectra contained Trp, C_α_-H, and N-C_α_-C bands; ** spectra did not contain Trp, C_α_-H, N-C_α_-C, and amide I bands but were not typical for amorphous carbon.

## Data Availability

Data is contained within the article or supplementary material.

## Supplementary Materials

The following supporting information can be downloaded at: https://www.mdpi.com/article/10.3390/bios13090895/s1, Figure S1: Flowchart of algorithm for processing the SERS maps of the NQ21 peptide, Figure S2: SERS spectra of the NQ21 peptide adsorbed on silver particles; SET 1—average SERS spectra built from each map, SET 2—average SERS spectra built from three maps, Figure S3: SERS spectra of the NQ21 peptide adsorbed on gold nanovoids; SET 1—average SERS spectra built from each map, SET 2—average SERS spectra built from three maps.
