# Supplementary material for: Comparative Study of SERS-Spectra of NQ21 Peptide on Silver Particles and in Gold-Coated “Nanovoids”
Source: Biosensors (Basel). 2023 Sep 20;13(9):895. doi: 10.3390/bios13090895 (PMC10526949; doi:10.3390/bios13090895)
Supplement: Supplementary file 1 [file biosensors-13-00895-s001.zip › biosensors-2533472-supplementary.pdf]

Comparative Study of SERS-Spectra of NQ21 Peptide on Silver Particles and in Gold-Coated "Nanovoids"

Siarhei Zavatski [1], Sergey Dubkov [2], Dmitry Gromov [2,3] and Hanna Bandarenka [1,*]

[1] Applied Plasmonic Laboratory, Belarusian State University of Informatics and Radioelectronics, 220013 Minsk, Belarus; s.zavatskij@bsuir.by
[2] Institute of Advanced Materials and Technologies, National Research University of Electronic Technology, Moscow 124498, Russia; svd@org.miet.ru (S.D.)
[3] Institute for Bionic Technologies and Engineering, I.M. Sechenov First Moscow State Medical University, Moscow 119435, Russia
* Correspondence: h.bandarenka@bsuir.by

Figure S1 shows flowchart of the algorithm for processing the SERS maps of the NQ21 peptide. Processing starts with entering the Raman shift range (1), in which the amide I band should be located. After uploading the SERS-spectra map (2), baseline correction is carried out for each spectrum (3) using the Asymmetric Least Squares Smoothing method [38] (4) and smoothing using the Savitzky-Golay method (5). After that, the Raman shift ranges are set to search additional bands corresponding to amorphous carbon, Trp, C_α-H and N-C_α-C (6). The analyzed spectrum is approximated by the Finite Difference method (FD) (7). There is a maximum in a given Raman shift range for amorphous carbon in the obtained first derivative of the spectrum (8). If this maximum is more than the set value (found empirically), then the spectrum corresponds to amorphous carbon and the transition to the analysis of the next spectrum is performed. Otherwise, the maximum of the first derivative is in the specified ranges for the Trp, C_α-H and N-C_α-C bands (9). If at least one of the maximum is less than the set value, then the analyzed spectrum is not the NQ21 SERS-spectrum and the transition to the next spectrum is performed. Otherwise, the maximum of the first derivative is in the specified range for the amide I band (10). If it is less than the set value, then the analyzed spectrum is the NQ21 SERS-spectrum, which does not contain the amide I band. Otherwise, the analyzed spectrum is the desired SERS-spectrum of the NQ21 peptide with the amide I band (11). The cycle ends after processing all spectra in the map (12). Results are saved (13), (14) and processing ends.

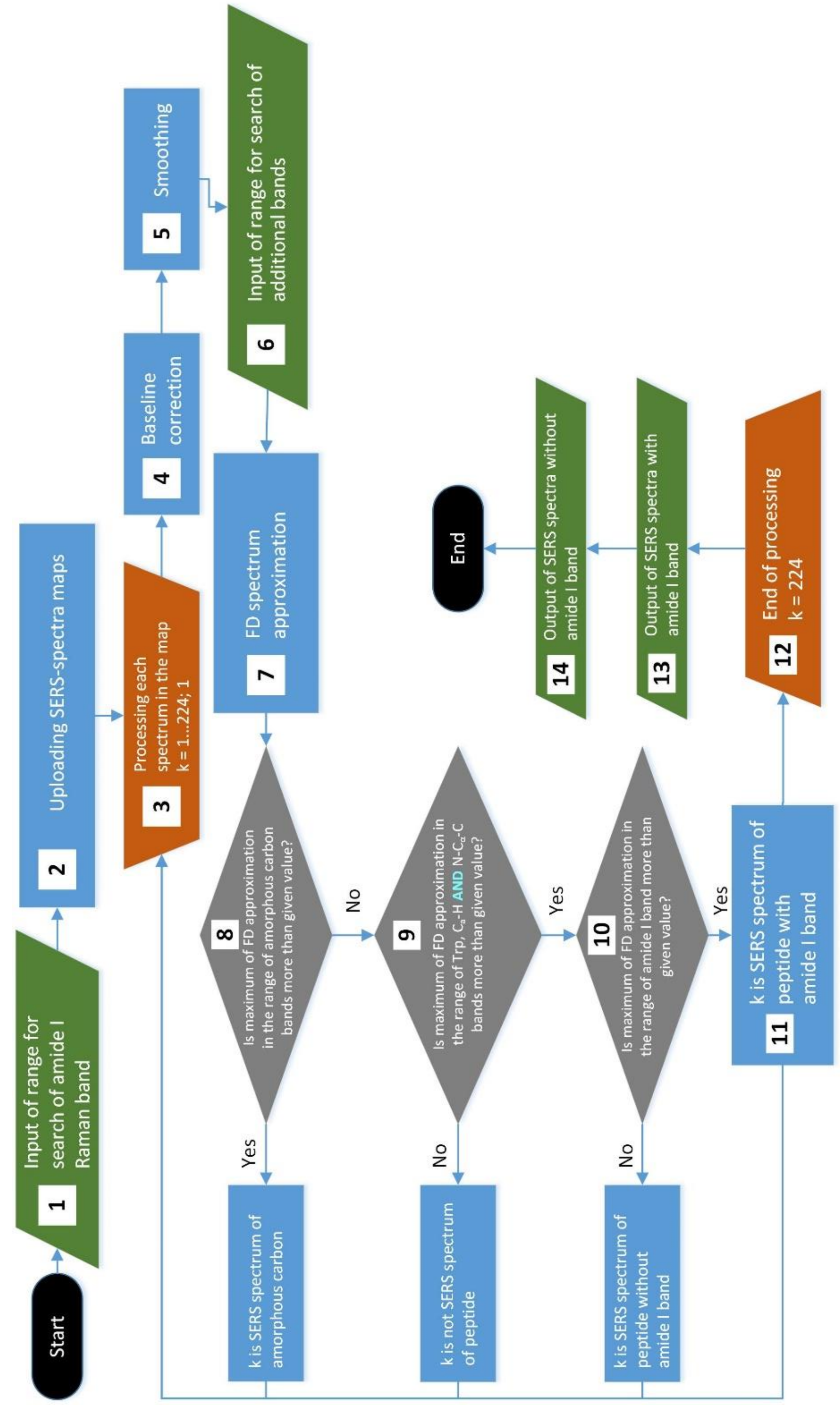

Figure S1. Flowchart of algorithm for processing the SERS maps of the NQ21 peptide.

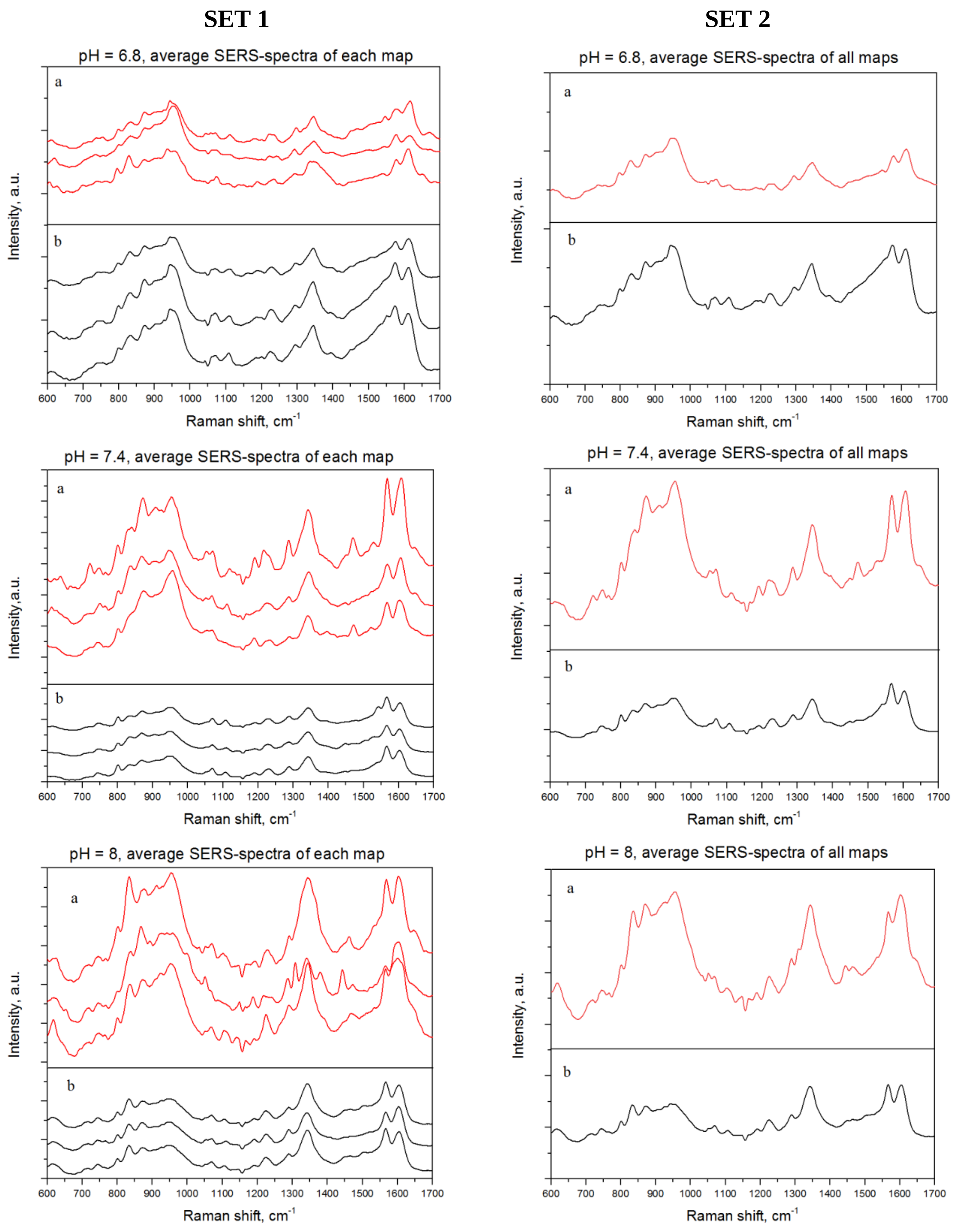

Figure S2. SERS-spectra of the NQ21 peptide adsorbed on silver particles. SET 1 – average SERS-spectra build from each map, SET 2 – average SERS-spectra built from three maps.

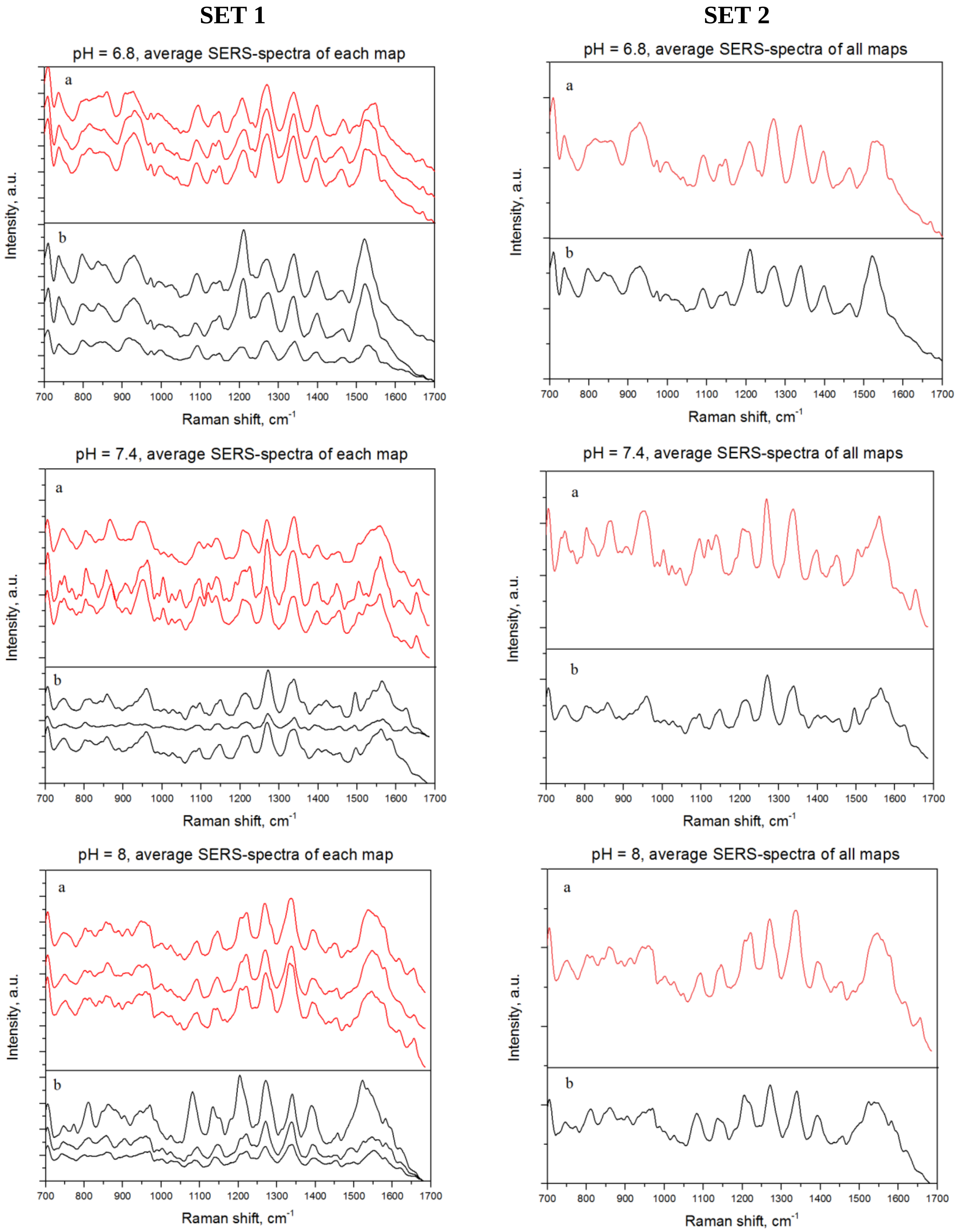

Figure S3. SERS-spectra of the NQ21 peptide adsorbed on gold nanovoids. SET 1 – average SERS-spectra build from each map, SET 2 – average SERS-spectra built from three maps.
